# Assessment of the Qualitative Fit Test and Quantitative Single-Pass Filtration Efficiency of Disposable N95 Masks Following Gamma Irradiation

**DOI:** 10.1001/jamanetworkopen.2020.9961

**Published:** 2020-05-26

**Authors:** Avilash Cramer, Enze Tian, Mitchell Galanek, Edward Lamere, Ju Li, Rajiv Gupta, Mike Short

**Affiliations:** 1 Harvard-MIT Division of Health Sciences and Technology, Boston, Massachusetts; 2 Department of Building Science, Tsinghua University, Beijing, China; 3 Department of Nuclear Science and Engineering, Massachusetts Institute of Technology, Cambridge; 4 Division of Neuroradiology, Massachusetts General Hospital, Boston

## Abstract

This quality improvement study examines the fit and filtration efficiency of disposable N95 masks after sterilization by cobalt-60 gamma irradiation.

## Introduction

The coronavirus disease 2019 pandemic has led to a dramatic shortage of masks and other personal protective equipment in hospitals around the globe. One component of personal protective equipment, the disposable N95 face mask, is in particular demand.^1,2^ To alleviate a shortage of N95 masks, many methods to resterilize them have been proposed and studied.^3^ Any method for resterilizing masks must not degrade the filtration efficiency of the mask.

This quality improvement study examines cobalt-60 gamma irradiation as a method of N95 mask sterilization. Viral inactivation of severe acute respiratory syndrome coronavirus has been reported at radiation doses of 10 kGy at most, with other studies supporting a radiation dose of 5 kGy for many types of viruses.^4,5^

Gamma irradiation has certain logistical advantages over other sterilization methods but there is a concern that radiation may damage the mask by cross-linking polymers within it and make them brittle. Ionizing radiation can disrupt the electrostatic charge distribution in the electret material of the mask and reduce its filtration efficiency against submicron particles.^6^

## Methods

This study was performed as part of hospital operations and infection control and, as per Massachusetts General Hospital policy, did not require institutional review board approval. This study follows the Standards for Quality Improvement Reporting Excellence (SQUIRE) reporting guideline.

A set of 3M 8210, 1805, and 9105 masks were irradiated using a cobalt-60 irradiator (GammaCell 220 E; Atomic Energy of Canada) at the Massachusetts Institute of Technology. Three masks of each type received 0 kGy (control), 1 kGy, 10 kGy, and 50 kGy of approximately 1.3 MeV gamma radiation from the source, at a dose rate of 2.2 kGy per hour.

The control (0 kGy) and 3 sets of irradiated masks (1, 10, and 50 kGy) were subjected to the Occupational Safety and Health Administration Gerson Qualitative Fit Test 50 (saccharin apparatus) by 1 of the authors (M.S.) and a Partners Healthcare physician in a blinded fashion. Another set of control and irradiated masks were tested for their particulate single-pass filtration efficiency. These masks were inserted into a specialized air duct, and ambient particulate matter was driven through the duct and the mask. The pressure differentials and flow velocities are shown in the Table. Three different particle sizes—0.3, 0.5, and 1 μm—were tested, and the single-pass filtration efficiency was measured using an optical particle counter (Aerotrak 9306; TSI Inc). The measurement system, which was not calibrated for N95 mask certification, was only used to assess the relative changes in the filtration efficiency.

**Table.** Single-Pass Filtration Efficiencies for Ambient Particles of Irradiated N95 Masks

Mask model and dose^a^	Single-pass filtration efficiency, mean (SD), %			Air pressure differential, Pa	Air flow velocity_, _m/s	Temperature, °C	RH, %
	0.3 μm	0.5 μm	1 μm				
9105, 0 kGy							
Mask 1	85.9 (3.9)	89.5 (4.5)	94.3 (4.1)	175.5	0.4	23.7	11.5
Mask 2	88.3 (3.5)	90.2 (3.6)	94.7 (3.6)	185.8	0.4	23.9	19.5
1805, 1 kGy							
Mask 1	29.2 (1.4)	41.1 (1.4)	70.3 (8.2)	186.1	0.1	23.9	20.4
Mask 2	31.1 (1.8)	43.0 (4.4)	72.1 (10.6)	186.6	0.1	23.8	20.6
9105, 10 kGy							
Mask 1	28.3 (1.1)	38.6 (2.5)	74.3 (8.1)	176.8	0.4	23.2	12.1
Mask 2	22.2 (1.1)	35.5 (3.2)	63.1 (11.1)	186.0	0.4	23.9	20.4
9105, 50 kGy							
Mask 1	24.8 (1.0)	36.7 (2.7)	69.5 (11.4)	176.8	0.4	23.1	12.4
Mask 2	23.8 (0.8)	35.1 (1.9)	72.0 (12.2)	184.2	0.4	23.9	20.2
8210, 0 kGy							
Mask 1	88.1 (1.9)	91.4 (1.8)	89.2 (2.7)	173.9	0.4	22.9	11.3
Mask 2	85.0 (2.2)	85.4 (1.7)	86.2 (3.5)	191.7	0.4	23.6	21.1
8210, 1 kGy							
Mask 1	30.8 (0.8)	43.1 (3.7)	75.0 (9.5)	185.0	0.4	23.7	21.2
Mask 2	26.2 (1.4)	37.4 (2.0)	61.6 (10.7)	187.1	0.4	23.7	21.4
8210, 10 kGy							
Mask 1	35.2 (0.9)	45.0 (2.8)	81.0 (5.7)	179.2	0.4	23.1	11.7
Mask 2	23.3 (1.6)	34.3 (2.5)	56.3 (13.0)	185.5	0.4	23.7	21.4
8210, 50 kGy							
Mask 1	28.2 (1.1)	36.0 (2.3)	66.0 (9.9)	186.1	0.4	23.6	21.4
Mask 2	29.6 (1.0)	45.2 (2.1)	71.9 (9.2)	186.4	0.4	23.7	21.2

Abbreviation: RH, relative humidity.

^a^ All masks were manufactured by 3M.

Statistical analyses were performed using R statistical software version 3.6.3 (R Project for Statistical Computing) with the level of significance set at *P* < .05. All tests were 2-sided. A linear mixed effects model was performed to assess the effects of dose (untreated, 1 kGy, 10 kGy, and 50 kGy) on the filtration efficiency of particles of 3 different sizes (0.3 μm, 0.5 μm, and 1 μm) using 2 masks per condition with 2 mask types (9105 and 8210). A Tukey honestly significant difference test was performed for the post-hoc analysis. Because of a lack of availability at the time of testing, we had to substitute the 1805 for the 9105 masks at 1 kGy. Data analysis was performed in April 2020.

## Results

Nine of 9 of the tested control and irradiated masks, when donned properly, passed the qualitative fit test. Single-pass filtration data are shown in the Table and in the Figure. There was statistically significant degradation of filtration efficiency for all treated masks. For example, for 1 of the 9105 masks, mean (SD) filtration efficiency for 0.3-μm particles decreased from 85.9% (3.9%) at 0 kGy to 28.3% (1.1%) at 10 kGy, and for 1 of the 8210 masks, mean (SD) filtration efficiency for 0.3-μm particles decreased from 88.1% (1.9%) to 30.8% (0.8%) at 1 kGy (for particle size, *F* = 59.0002; for radiation dose, *F* = 75.6986; *P* < .001 for both). However, there was no difference in filtration efficiency between the masks irradiated at1 kGy (mean [SE] estimate, 42.275 [3.542]), 10 kGy (mean [SE] estimate, 44.258 [23.542]), or 50 kGy (mean [SE] estimate, 44.117 [3.542]). For example, for 1 of the 8210 masks, the mean (SD) filtration efficiency for 0.3-μm particles was 26.2% (1.4%) at 1 kGy, 23.3% (1.6%) at 10 kGy, and 29.6% (1.0%) at 50 kGy. The filtration efficiency for 1-μm particles was greater than that for 0.5-μm particles (mean [SE] estimate, 23.125 [3.068]; *z* = 7.538; *P* < .001) which, in turn, was greater than that for 0.3-μm particles (mean [SE] estimate, 9.219 [3.068]; *z* = 3.005; *P* = .007).

**Figure.**
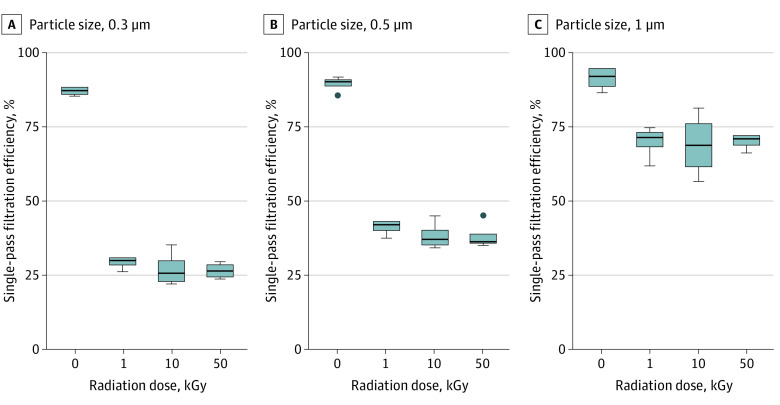
Single-Pass Filtration Efficiency of N95 Masks That Underwent Gamma Irradiation Box plots show data from a given particulate size for N95 masks that received 0, 1, 10, and 50 kGy gamma radiation doses from a cobalt-60 source. Tops and bottoms of boxes denote 75th and 25th percentiles, respectively. Lines within boxes denote medians. Circles denote outliers. Error bars were calculated by 6 observations of the upstream and downstream particle concentration.

## Discussion

This study has limitations. The test we used to assess filtration efficiency is not approved by the National Institute for Occupational Safety and Health, and particulate matter smaller than 0.3 μm was not examined. The number and type of masks studied was limited by the current supply shortage. However, these findings suggest that a qualitative fit test alone is unable to fully assess mask integrity and that at the doses required for sterilization, gamma radiation degrades the filtration efficiency of N95 masks.
